# Supplementary material for: T-cell receptors identified by a personalized antigen-agnostic screening approach target shared neoantigen KRAS Q61H
Source: Front Immunol. 2025 Mar 17;16:1509855. doi: 10.3389/fimmu.2025.1509855 (PMC11955635; doi:10.3389/fimmu.2025.1509855)
Supplement: Supplementary file 8 [file DataSheet1.pdf]

Table S1

Table S1: Clinical course of patient 3

Time (mo, yr)	Diagnosis, clinical findings	Treatment	Staging information	Molecular Pathology
**Jun 2018**	lung adeno-carcinoma, left upper lobe	lobectomy left upper lobe and mediastinal lymph node dissection	pT2a pN0 (0/24) pL1 pV1 R0 cM0;<br>UICC IB	PD-L1 55% (TPS)
**Jul-Sep 2019**	local recurrence	chemoradiotherapy (carboplatin, vinorelbine; total irradiation dose 66 Gy)	-	-
**Oct 2019-Nov 2020**	-	durvalumab maintenance	-	-
**Jan 2021**	progressive local recurrence, no evidence of metastatic disease	extended left pneumonectomy	yrpT2a yrpN0 (0/11); regression grade IIa (≥ 10% vital tumor tissue)*	*KRAS* c.183A>T/p.Q61H; *TP53* c.638G>A/p.R213Q; *SMO* c.1225G>T/p.G409C; PD-L1 50-60% (TPS)
**Since 2021 until now**	No evidence of disease			

*: according to Junker et al., doi: 10.1378/chest.120.5.1584

Figure S1

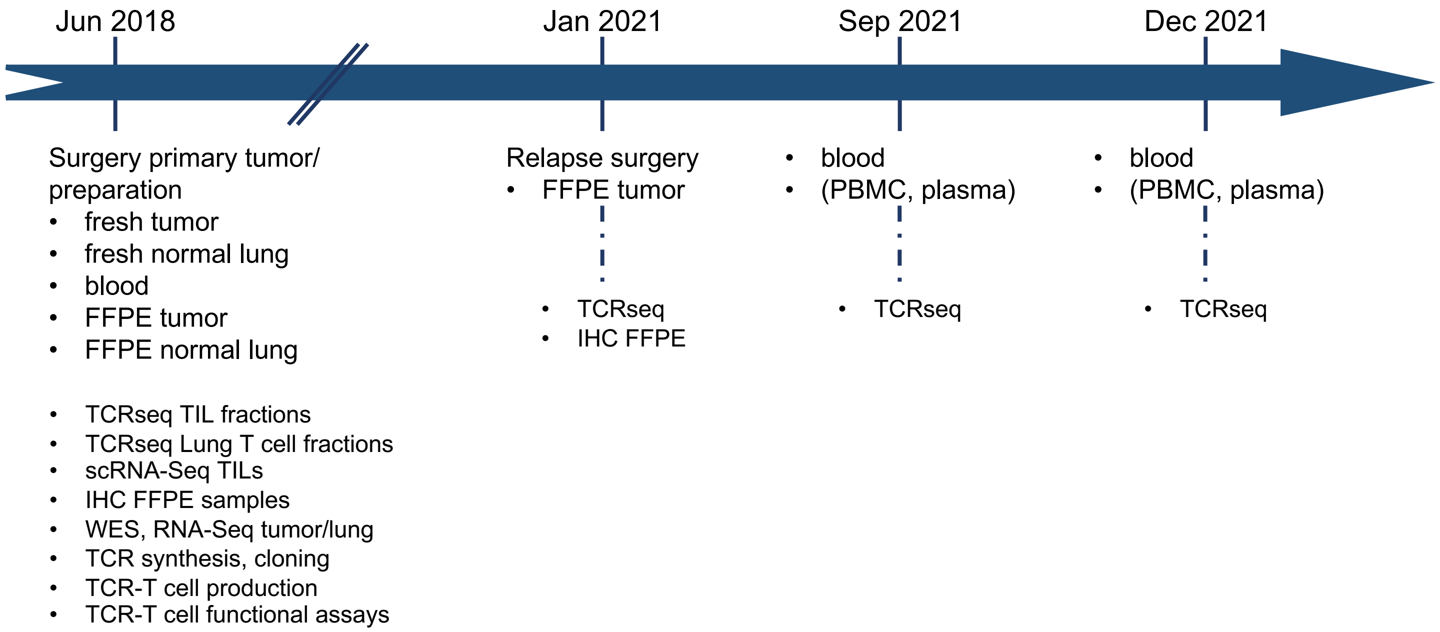

Fig. S1: Patient 3 clinical data, samples collected, analyzed and methods applied. IHC, Immunohistochemistry; FFPE, formaline-fixed paraffin-embedded tissue; WES, whole exome sequencing; scRNA-Seq, single cell RNA-sequencing; TCRseq, TCR-VDJ-sequencing.

Figure S2: IHC panel primary tumor and relapse

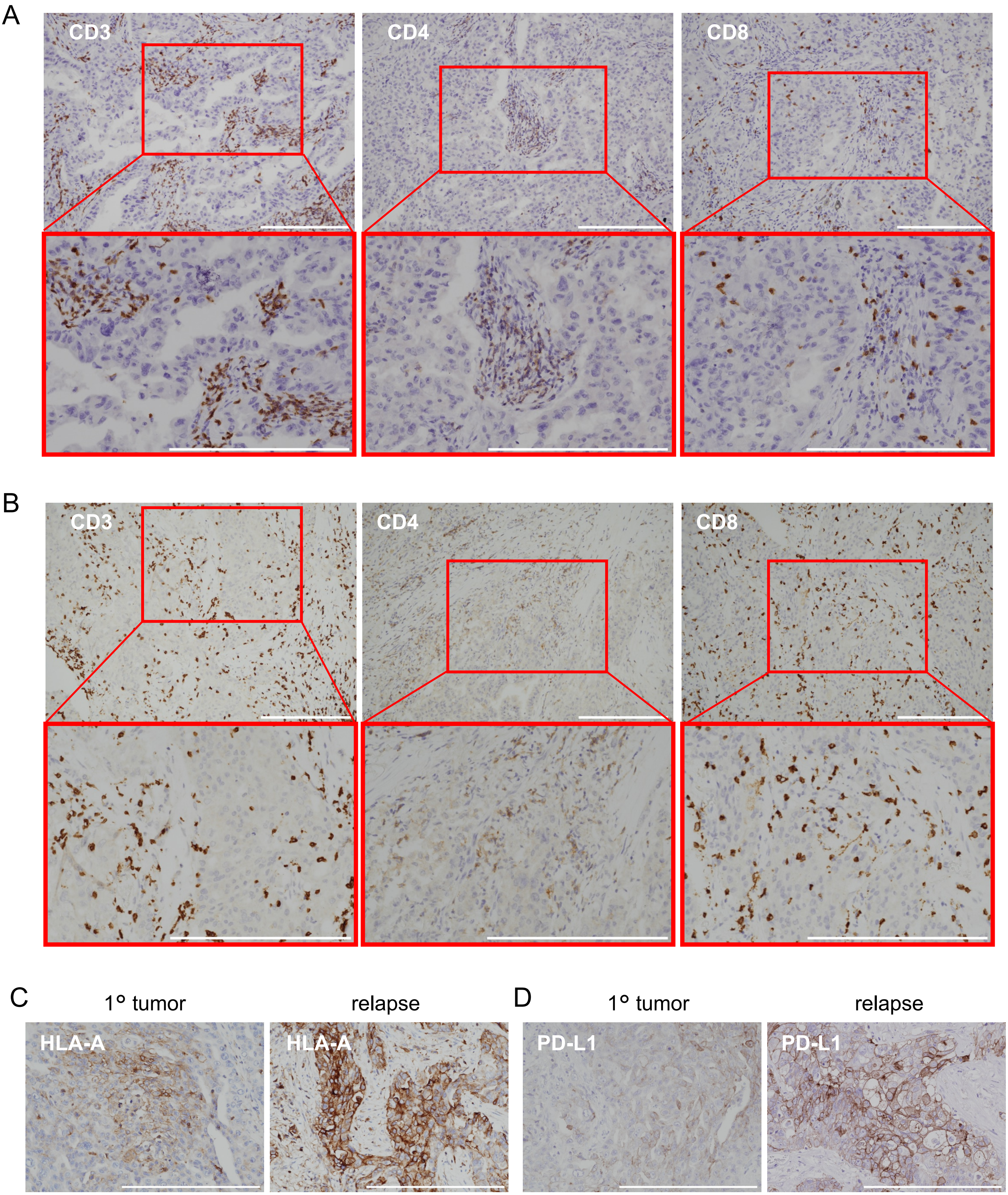

Fig. S2: Immunohistochemistry (IHC) of FFPE slices from the primary tumor of patient 3 (A) and the tumor relapse surgically removed 32 months later (B). Primary tumor and relapse expressed HLA-A- (C) and PD-L1 (D). Samples were stained with antibodies against the indicated cell surface proteins. (Scale bars: 50 µm) Pictures were taken at nominal magnification of 40x and 80x, respectively.

Figure S3

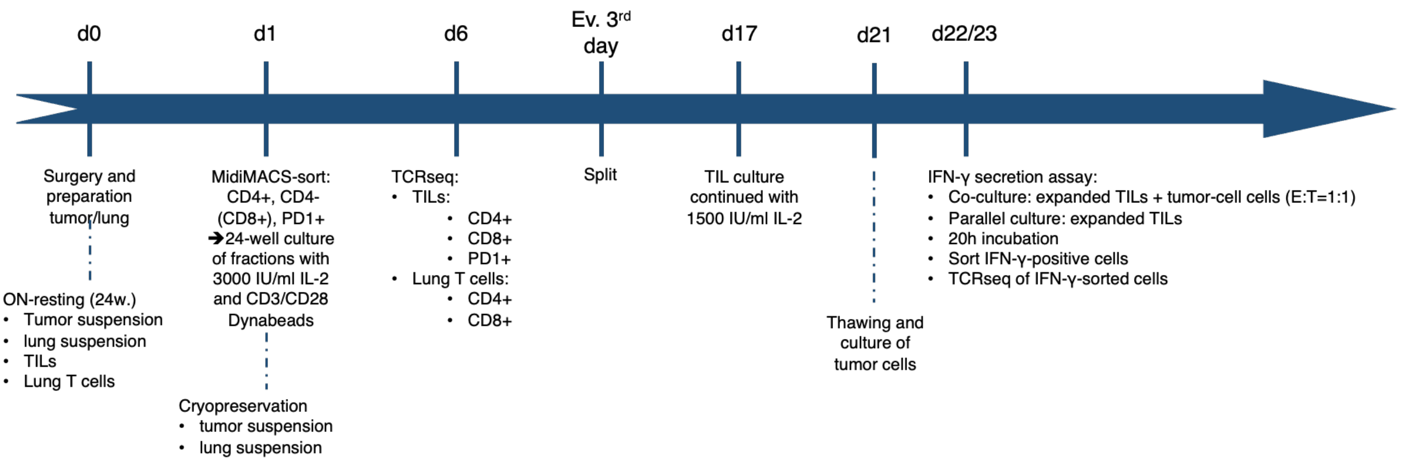

Fig. S3: Outline and timeline of patient 1 experiments including surgical specimen preparation, MidiMACS-fractionation of TILs and lung-infiltrating lymphocytes, expansion of TILs using an in-house protocol with high-dose IL-2 and CD3/CD28 dynabeads, TCRseq at day 6 and after TIL-expansion and challenge with autologous tumor cells (d23). For procedural reasons, the TCRseq at day 1 (baseline, ex vivo) could not be performed. The expansion for five days can have changed the composition of the TILs and lung-resident T cells. However, both cultures were run in parallel and the candidate tumor-specific T-cell clonotypes were predicted based on comparative TCRseq between both cultures treated in the same way. To test their tumor-reactivity, the expanded TILs were tested by IFN-γ Secretion Assay – Cell Enrichment and Detection Kit (Miltenyi). As per protocol, half of the expanded TILs were stimulated with tumor cells and the other half cultured in the same way without antigen-challenge. After 20h, both cell cultures were treated with IFN-γ catch reagent and IFN-γ detection antibody and positive cells isolated by magnetic cell enrichment. Isolated cells were subjected to TCRseq. From the control reaction, no IFN-γ stained lymphocytes were obtained. The TCR repertoire analysis of the tumor-stimulated CD8 T cells is shown in Figure 1A.

Figure S4

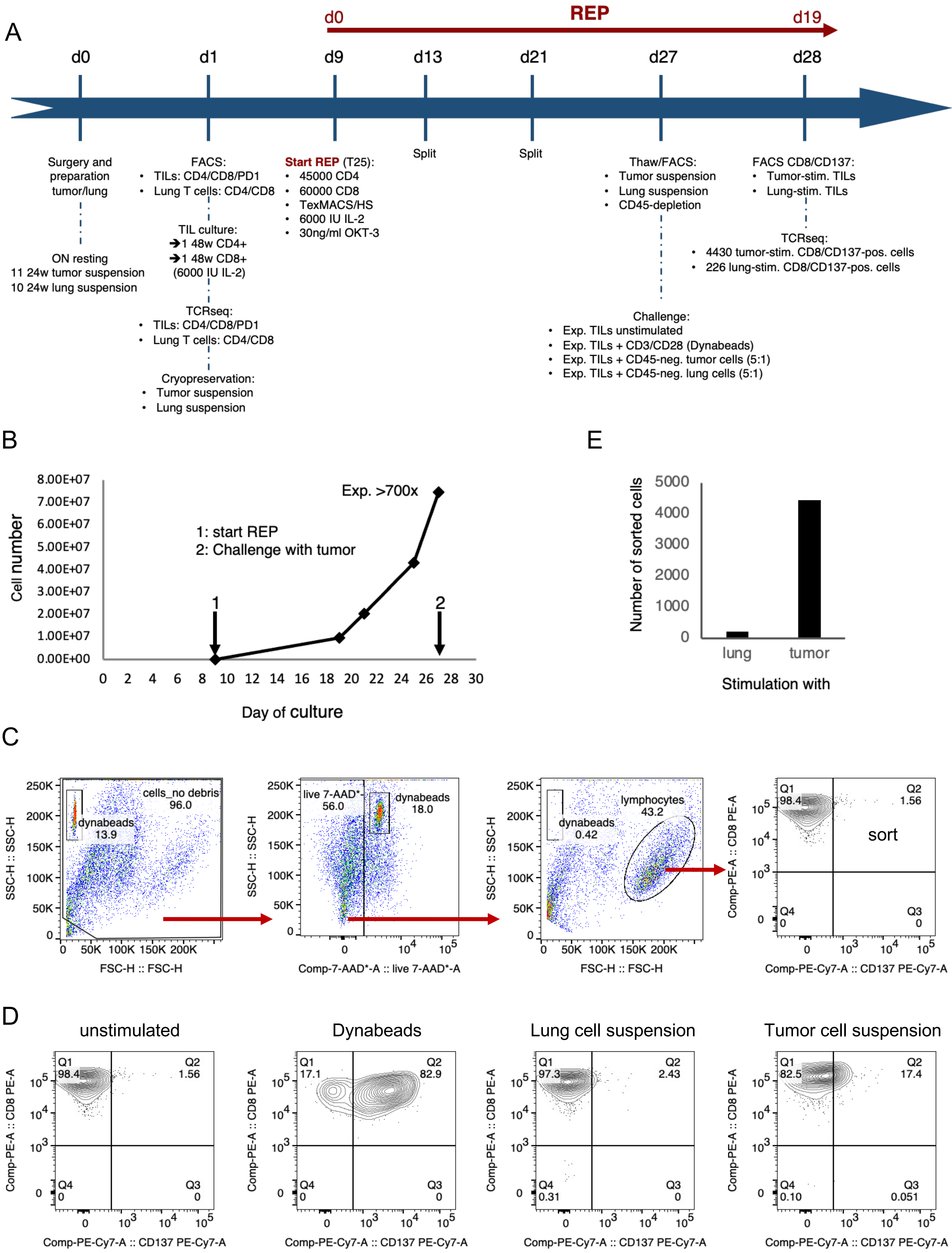
A
d0
REP
d19
d0
d1
d9
d13
d21
d27
d28
Surgery and preparation tumor/lung
ON resting
• 11 24w tumor suspension
• 10 24w lung suspension
FACS:
• TILs: CD4/CD8/PD1
• Lung T cells: CD4/CD8
TIL culture:
→1 48w CD4+
→1 48w CD8+
(6000 IU IL-2)
TCRseq:
• TILs: CD4/CD8/PD1
• Lung T cells: CD4/CD8
Cryopreservation:
• Tumor suspension
• Lung suspension
Start REP (T25):
• 45000 CD4
• 60000 CD8
• TexMACS/HS
• 6000 IU IL-2
• 30ng/ml OKT-3
Split
Split
Thaw/FACS:
• Tumor suspension
• Lung suspension
• CD45-depletion
Challenge:
• Exp. TILs unstimulated
• Exp. TILs + CD3/CD28 (Dynabeads)
• Exp. TILs + CD45-neg. tumor cells (5:1)
• Exp. TILs + CD45-neg. lung cells (5:1)
FACS CD8/CD137:
• Tumor-stim. TILs
• Lung-stim. TILs
TCRseq:
• 4430 tumor-stim. CD8/CD137-pos. cells
• 226 lung-stim. CD8/CD137-pos. cells
B
Cell number
8.00E+07
7.00E+07
6.00E+07
5.00E+07
4.00E+07
3.00E+07
2.00E+07
1.00E+07
0.00E+00
Exp. >700x
1: start REP
2: Challenge with tumor
1
2
0 2 4 6 8 10 12 14 16 18 20 22 24 26 28 30
Day of culture
E
Number of sorted cells
5000
4000
3000
2000
1000
0
lung
tumor
Stimulation with
C
SSC-H :: SSC-H
cells_no debris
96.0
dynabeads
13.9
FSC-H :: FSC-H
live 7-AAD*-
56.0
dynabeads
18.0
Comp-7-AAD*-A :: live 7-AAD*-A
lymphocytes
43.2
dynabeads
0.42
FSC-H :: FSC-H
Comp-PE-A :: CD8 PE-A
Q1
98.4
Q2
1.56
sort
Q4
0
Q3
0
Comp-PE-Cy7-A :: CD137 PE-Cy7-A
D
unstimulated
Q1 98.4
Q2 1.56
Q4 0
Q3 0
Dynabeads
Q1 17.1
Q2 82.9
Q4 0
Q3 0
Lung cell suspension
Q1 97.3
Q2 2.43
Q4 0.31
Q3 0
Tumor cell suspension
Q1 82.5
Q2 17.4
Q4 0.10
Q3 0.051
Comp-PE-A :: CD8 PE-A
Comp-PE-Cy7-A :: CD137 PE-Cy7-A

Fig. S4: Preparation of surgical samples of patient 2, FACS-fractionation of TILs and lung-infiltrating lymphocytes, expansion of TILs using a protocol adapted from the „small scale rapid expansion protocol" (REP) reported by Donia et al. (Scand J Immunol 2012 Vol. 75 Issue 2 Pages 157-67.), and TCRseq at baseline (d1) and after TIL-expansion and challenge with autologous tumor cells (d28). (A) Outline and timeline of the procedure. (B) Expansion of the TILs by REP. (C) Gating strategy for the analysis of the expanded TILs challenged with tumor cells or normal lung suspension. (D) FACS plots showing the challenge reactions and controls (TILs unstimulated and challenged with Dynabeads). (E) Sorting result of tumor and lung-stimulated and sorted (CD8/CD137-positive) cells. From the few lung-stimulated TILs not enough gDNA could be isolated for TCRseq. The TCR repertoire analysis of the tumor-stimulated CD8 T cells is shown in Figure 1B.

Table S2

Score	TRB-CDR3 peptide	Freq. tumor	Freq. lung	Tumor / lung	Freq. PD1+	Selected	Tumor-reactive[1]
1	CASSVDRGAEAFF	3.6602	0.4070	8.9903	2.0895	+	+ (2.7417)
2	CAWNKQVDGYTF	2.3939	0.0546	43.7316	0.8854	+	+ (0.7399)
3	CASSFGVMNTEAFF	2.3298	1.4606	1.5951	0.5585		0.0893
4	CASSPDGETQYF	2.2556	0.2012	11.2029	0.8427	+	+ (1.1265)
5	CASSLGQAYEQYF	1.7864	1.0162	1.7578	0.1642		0.0449
6	CASSPVAGMNTEAFF	1.4172	3.3855	0.4186	0.2980		+ (0.2441)
7	CAISDWTGSNYGYTF	1.1625	0.0583	19.9125	0.2638	+	+ (2.3817)
8	CASSGRGDLLEQYF	1.1208	0.5964	1.8788	0.0858		0.0000
9	CASSETGAAETQYF	1.0946	4.8834	0.2241	0.2134		0.0971
10	CASSRLAGGTDTQYF	0.9505	2.4768	0.3837	0.0923		0.0399
11	CASSSGLVYEQYF	0.8981	0.1284	6.9893	0.4463	+	+ (0.8880)
12	CASSTGTGGLGELFF	0.8748	0.1020	8.5688	0.7057	+	+ (0.6506)
13	CASSEAPPLYYEQYF	0.8549	0.2113	4.0447	0.0000		0.0000
14	CASSNDRAGLNEQFF	0.8461	0.7394	1.1442	0.0240		0.0000
15	CATSDGRLEQFF	0.8219	0.1129	7.2728	0.8480	+	0.0000
16	CASSLGYRYGTEAFF	0.8103	3.5121	0.2307	0.2943		0.0000
17	CASSQDNGGYGYTF	0.8030	0.1484	5.4062	0.1943	+	0.0000
18	CASSQGDSFYGYTF	0.8001	0.1949	4.1037	0.0394		0.0000
19	CASSADLGDRVNGYTF	0.7821	0.8068	0.9693	0.2459		0.0000
20	CASSLDRGGYEQYF	0.7540	0.1402	5.3728	0.1224		0.0000

[1] IFN-γ-positive captured cells after stimulation of expanded TILs with autologous tumor cells. Positivity threshold: 0.1%.

Table S2: Top 20 TIL clonotypes of patient 1: Frequencies (percentages) of the top 20 TILs in comparison to additional fractions of the primary tumor and after expansion and tumor challenge.

Table S3

Score	TRB-CDR3 peptide	Freq. tumor	Freq. lung	Tumor / lung	Freq. PD1+	Selected	Tumor-reactive[1]
1	CASSLGTAGEQFF	6.310	0.296	21.35	3.520	+	+ (3.443)
2	CAISGLLDAGDPDTQYF	2.990	0.052	57.29	1.743	+	+ (0.215)
3	CASSQEPVSSYNSPLHF	2.393	0.128	18.75	1.001	+	+ (3.431)
4	CASSRTGFEANTEAFF	2.011	1.040	1.93	1.248	-	0.000
5	CASSQGDRGRENSPLHF	1.882	0.602	3.12	0.446	-	+ (21.431)
6	CASSPGQGDYEQYF	1.796	0.063	28.46	1.055	+	0.000
7	CASSLWTSADTQYF	1.796	29.810	0.06	0.151	-	+ (5.235)
8	CAWSGGQGRLNQPQHF	1.639	0.205	8.00	0.193	+	+ (0.390)
9	CASSALGDTEAFF	1.371	0.000	>1000	0.144	+	0.000
10	CASSIIGTGDQYF	1.314	0.086	15.29	0.258	+	0.000
11	CAISTVEADTIYF	1.309	0.005	276.10	0.640	+	0.000
12	CASSYLTDTQYF	1.172	0.748	1.56	0.731	-	0.000
13	CASSPWTDGGYTF	1.104	0.253	4.36	0.052	-	0.000
14	CASSWGTTSPLHF	1.060	0.049	21.48	0.241	+	0.000
15	CASSPTTGDEQYF	1.015	0.042	24.32	0.835	+	0.000
16	CAWEPGHLGETQYF	0.981	0.050	19.68	0.056	-	0.079
17	CASSPVGGYTEAFF	0.979	0.572	1.71	0.054	-	0.000
18	CASSIGKTAYEQYF	0.776	0.019	40.90	0.000	-	+ (2.639)
19	CASSLGPAGNTIYF	0.758	0.106	7.12	0.111	-	+ (3.547)
20	CASSSLGQPNTEAFF	0.663	0.003	199.83	0.401	-	0.000

[1] CD137-positive sorted cells after stimulation of expanded TILs with autologous tumor cells. Positivity threshold: 0.1%.

Table S3: Top 20 TIL clonotypes of patient 2: Frequencies (percentages) of the top 20 TILs in comparison to additional fractions of the primary tumor and after expansion and tumor challenge.

Figure S5

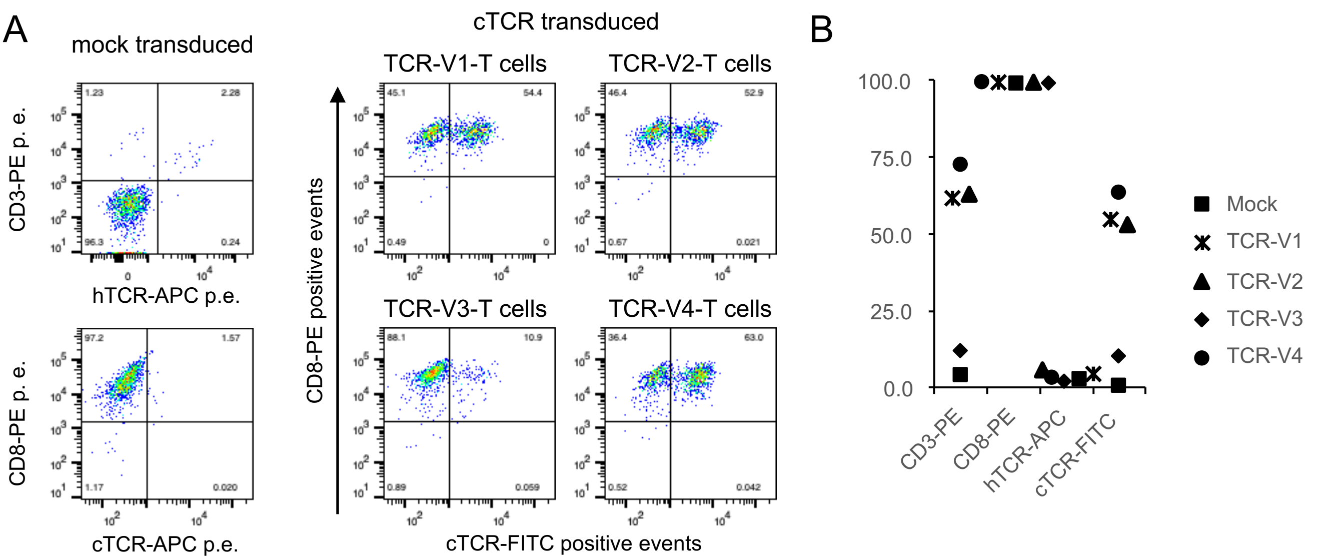

Fig. S5: Flow cytometry analysis of donor CD8 T cells transduced with tumor-specific TCR-constructs. (A) Endogenous (human) hTCR-KO efficiency (left) and expression of recombinant chimeric (c)TCRs engineered with murine Trac and Trbc domains (middle) is shown. Human and murine constant domains are detected by specific antibodies. Because CD3 and TCR expression correlate, mock-transduced hTCR-KO cells were CD3-negative while maintaining expression of CD8. Endo TCR-KO was >97%. (B) TCR-T cells transduced with TCR-V1, V2, and V4 showed high expression of cTCRs (range: 53-63%); TCR-V3-T cells showed only 11% cTCR expression and were excluded from subsequent response analysis.

Figure S6

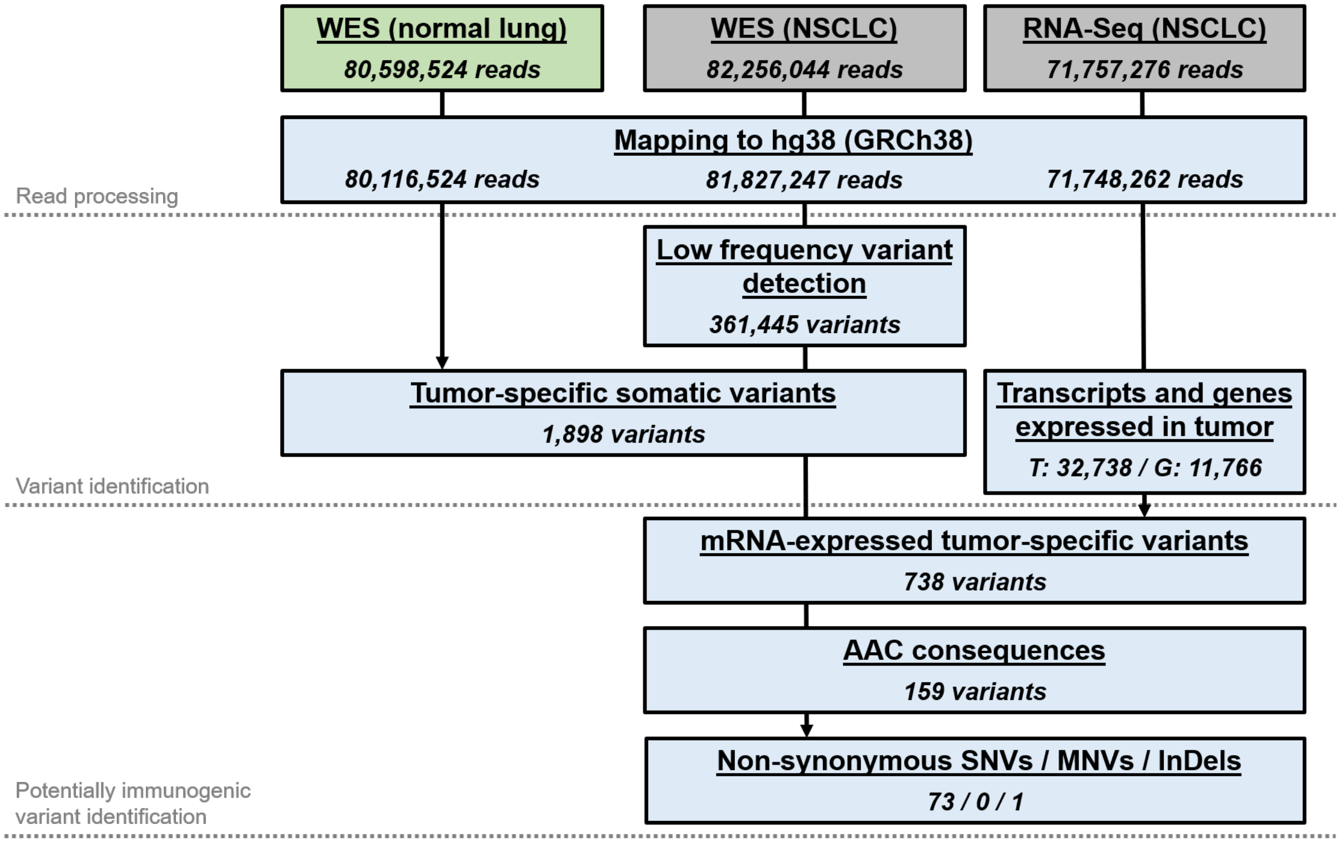

Fig. S6: Overview of the exome- and transcriptome (RNA-seq) analyses pipelines using CLC Genomics Workbench tools. For clarity, the display of apps used for raw read preparations, quality assessments, annotations and comparisons of datasets included in the respective workflows were omitted. Numbers and labels at the bottom of the boxes detail the results of each analysis step.

Figure S7

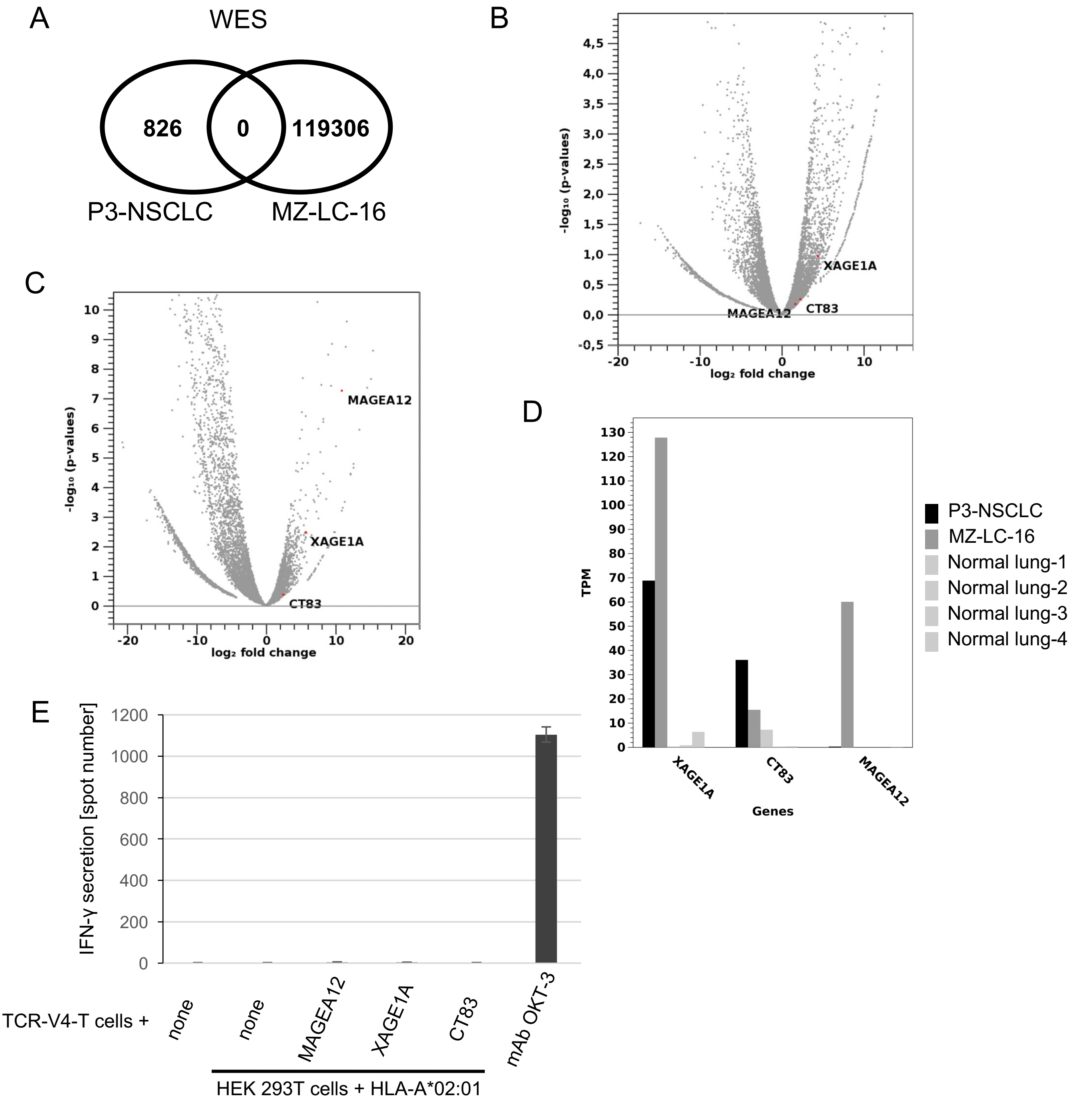

Fig. S7: Screening for target antigen candidates of TCR-V4-T cells. (A) Whole exome sequencing and quality filtering discovered 826 non-synonymous variants in patient 3's NSCLC tissue and 119306 in MZ-LC-16 cells. SNVs compared to reference genome Hg38 in patient 3 normal lung were subtracted from autologous tumor variants. (For MZ-LC-16 cells no normal control was available.) No non-synonymous SNVs, MNVs, or InDels were shared between patient 3 NSCLC and MZ-LC-16, and thus, no shared neoantigen candidates were found. (B-D) Because no autologous normal tissue was available for cell line MZ-LC-16, differential gene expression (DGE) analysis of both, MZ-LC-16 and patient 3 NSCLC, was performed by comparison with gene expression profiles of four normal lung samples (defined as a group). To select for overexpression of tumor-associated antigens, the DGE-data were filtered for overlap with 141 published Cancer/Germline genes frequently expressed in NSCLC. Only C/G genes MAGEA12, XAGE1A, and CT83 were overexpressed in patient 3's NSCLC (B) and MZ-LC-16 (C). (D) Transcripts per million reads (TPM) of the three candidate TAA in both tumor samples and the four normal lung samples. cDNAs encoding the longest transcripts of the three genes were cloned into expression vectors, co-transfected with HLA-A*02:01 into HEK293T cells and the cells tested for recognition by TCR-V4-expressing TCR-T cells via ELISpot (E). No recognition was detected. Treatment with mAb OKT-3 was used as positive control of TCR-T cells activation.

Figure S8

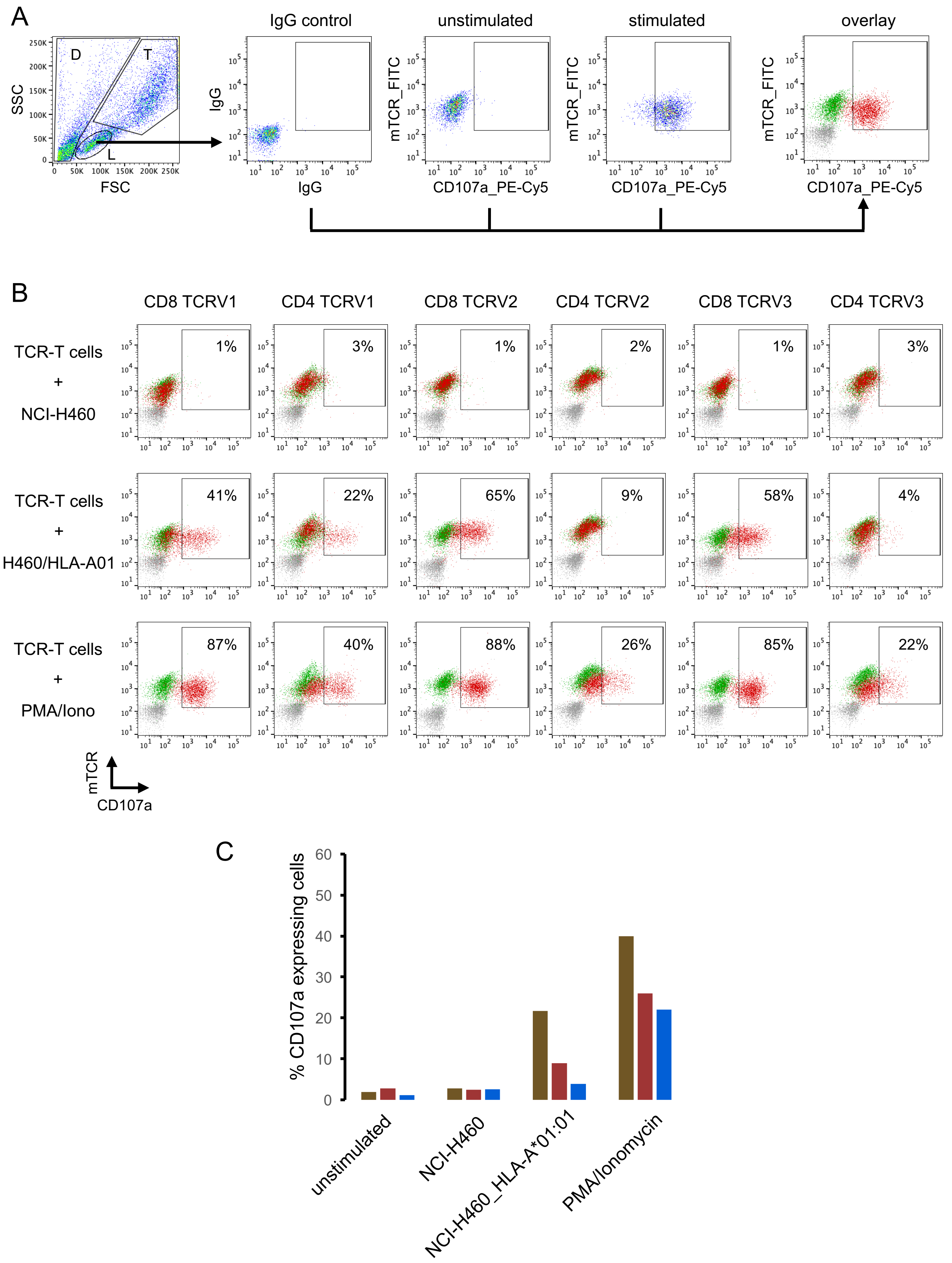
A
IgG control
unstimulated
stimulated
overlay
SSC
FSC
D
T
L
IgG
mTCR_FITC
CD107a_PE-Cy5
B
CD8 TCRV1
CD4 TCRV1
CD8 TCRV2
CD4 TCRV2
CD8 TCRV3
CD4 TCRV3
TCR-T cells + NCI-H460
1%
3%
1%
2%
1%
3%
TCR-T cells + H460/HLA-A01
41%
22%
65%
9%
58%
4%
TCR-T cells + PMA/Iono
87%
40%
88%
26%
85%
22%
mTCR
CD107a
C
% CD107a expressing cells
unstimulated
NCI-H460
NCI-H460_HLA-A*01:01
PMA/Ionomycin

Figure S8

Fig. S8: CD107a expression indicates activation-induced degranulation of mutated KRAS-specific TCR transduced TCR-T cells as a surrogate for cytolytic activity of the T cells. (A) Gating strategy: In the scatter plot, lymphocytes (L), viable tumor cells (T), dead cells and debris (D) can be distinguished by cell size and granularity. Lymphocytes were analyzed regarding cellular autofluorescence (Ab isotype control), staining of unstimulated lymphocytes (TCR-positive/CD107a-negative), and lymphocytes stimulated with PMA/Ionomycin (example shown), epitope-negative NCI-H460 and epitope-presenting NCI-H460/HLA-A*01:01. The results are displayed as overlays of all reactions. (B) Tested CD8-positive and CD4-positive TCR-T cell populations are shown on top of the columns. Stimulation conditions are shown on the left. Results are CD107a-positive cells (red) shown as percentages of all lymphocytes. (C) Box plot showing the percentages of degranulating CD4-positive TCR-T cells (CD107a-positive) for each stimulation condition. Colors code for the TCRs the lymphocytes were transduced with: TCR-V1 (brown), TCR-V2 (red), TCR-V3 (blue). The corresponding result for CD8-positive TCR-T cells is shown in Figure 3F.

Figure S9

A

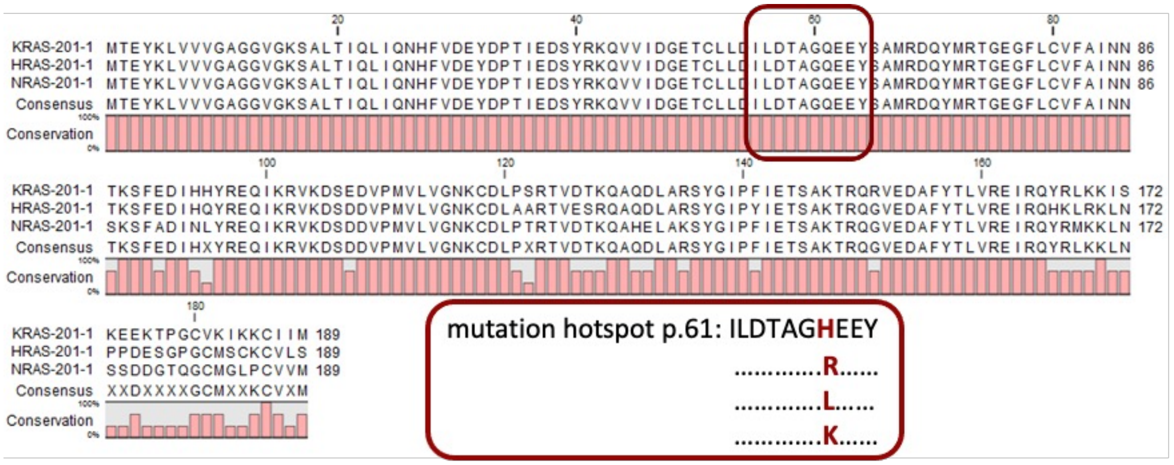

B

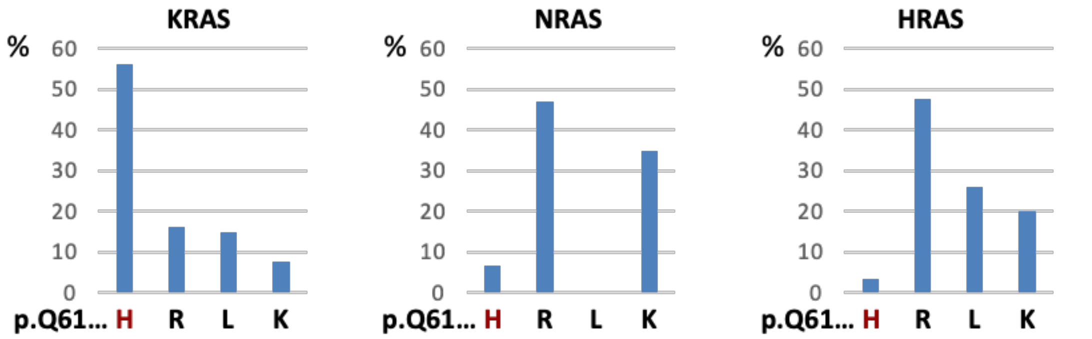

PAAD > COAD > MM > LUAD MM > SKCM > COAD > THCA BLCA > HNSC > THCA > PCPG

Fig. S9: Amino acid position 61-mutations in Ras family isoforms. (A) Alignment of K-, H-, and NRAS highlighting the peptide coding regions of hotspot mutations at position 61. (B) Q61 hotspot mutations in RAS family proteins and their prevalence in different tumor types according to COSMIC database. PAAD (Pancreatic adenocarcinoma), COAD (Colon adenocarcinoma), MM (Multiple Myeloma), LUAD (Lung adenocarcinoma), SKCM (Skin cutaneous melanoma), THCA (Thyroid carcinoma), BLCA (Bladder Urothelial Carcinoma), HNSC (Head and Neck squamous cell carcinoma), PCPG (Pheochromocytoma and Paraganglioma).

Figure S10

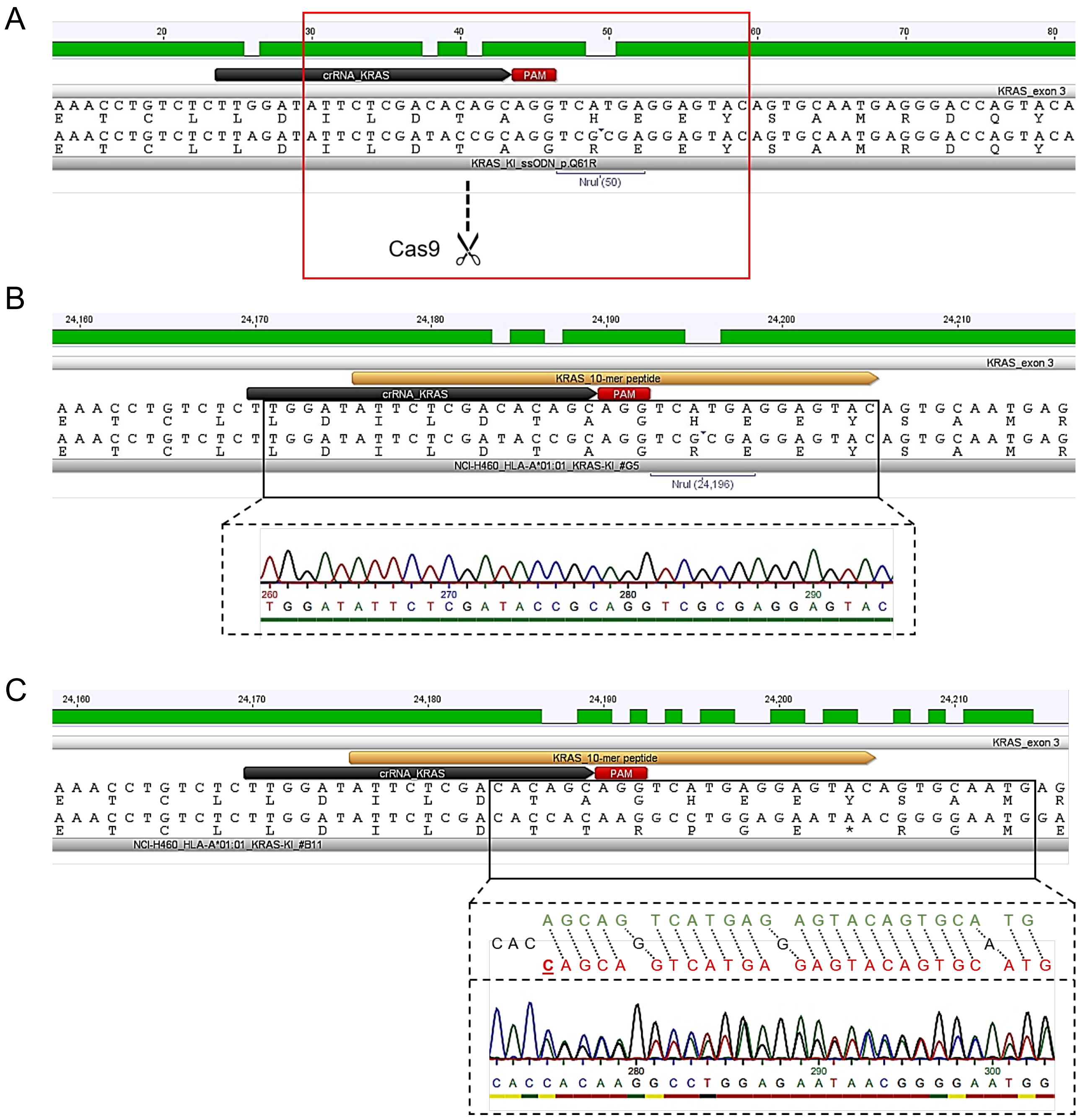

Fig. S10: CRISPR/CAS9 genetic engineering strategy and results showing the replacement of the mutation Q61H by Q61R in KRAS. (A) KRAS crRNA and CAS9 cutting site. (B) Alignment of the KRAS target sequence and the engineered sequence in NCI-H460/HLA-A*01:01 clone G5. (C) Alignment of the KRAS target sequence and the engineered sequence in NCI-H460/HLA-A*01:01 clone B11.

Figure S11

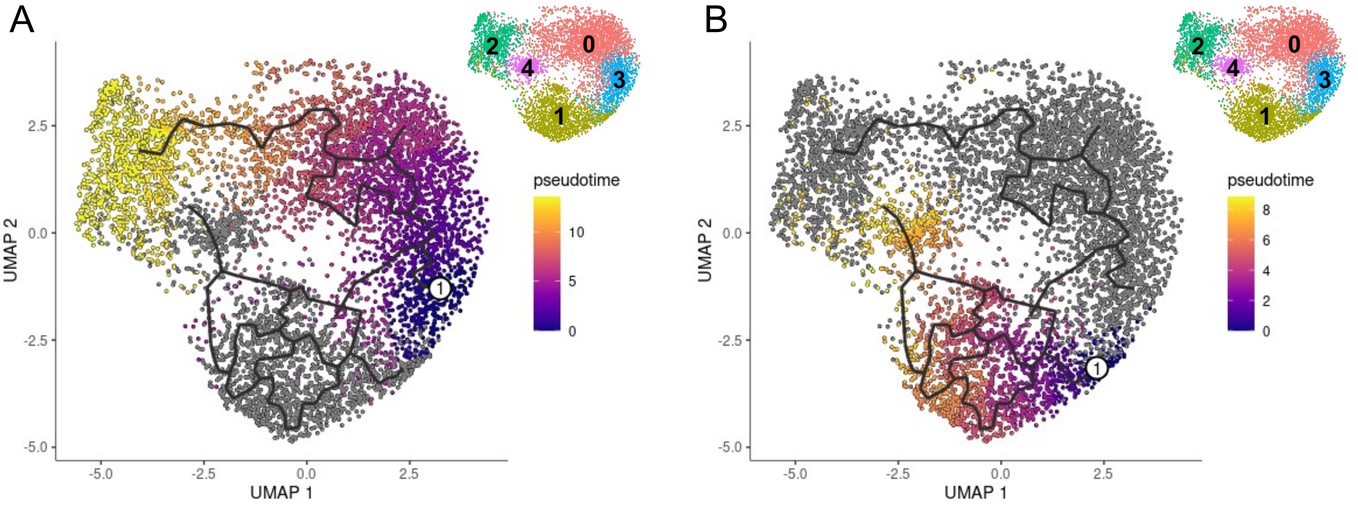

Fig. S11: Differentiation trajectory analysis of the CD8 T-cell subclustering (insets showing cluster numbering). UMAP visualization of two partitions, analyzed by Monocle 3 to calculate two distinct pseudotime trajectories. (A) The first partition was derived from the clusters enriched for predicted tumor-specific clonotypes (clusters 0, 2 and 3; Fig. 6D). The trajectory was rooted at cluster 3 containing the least differentiated clonotypes, as indicated by “1”. This trajectory passes through cluster 0 to the terminally differentiated, exhausted cluster 2, which is highlighted in yellow with the maximum pseudotime. (B) The second partition is made out of clusters 1 and 4, which are associated with predicted bystander T-cells (Fig. 6E). The root of this trajectory was set close to cluster 3 (”1”) representing the least differentiated clonotypes and ended at the cytotoxic cluster 4, with the greatest pseudotime displayed in yellow. While the differentiation trajectory in (A) is well in line with tumor experienced chronically stimulated and exhausted TIL clonotypes, the second trajectory (B) is characteristic of tissue-resident “bystander” T cells.
